# LERCause: Deep learning approaches for causal sentence identification from nuclear safety reports

**DOI:** 10.1371/journal.pone.0308155

**Published:** 2024-08-22

**Authors:** Jinmo Kim, Jenna Kim, Aejin Lee, Jinseok Kim, Jana Diesner

**Affiliations:** 1 School of Information Sciences, University of Illinois Urbana-Champaign, Champaign, Illinois, United States of America; 2 School of Information, Florida State University, Tallahassee, Florida, United States of America; 3 Institute for Social Research, University of Michigan, Ann Arbor, Michigan, United States of America; 4 School of Information, University of Michigan, Ann Arbor, Michigan, United States of America; 5 School of Social Sciences and Technology, Technical University of Munich, Munich, Germany; European Commission, ITALY

## Abstract

Identifying causal sentences from nuclear incident reports is essential for advancing nuclear safety research and applications. Nonetheless, accurately locating and labeling causal sentences in text data is challenging, and might benefit from the usage of automated techniques. In this paper, we introduce LERCause, a labeled dataset combined with labeling methods meant to serve as a foundation for the classification of causal sentences in the domain of nuclear safety. We used three BERT models (BERT, BioBERT, and SciBERT) to 10,608 annotated sentences from the Licensee Event Report (LER) corpus for predicting sentence labels (Causal vs. non-Causal). We also used a keyword-based heuristic strategy, three standard machine learning methods (Logistic Regression, Gradient Boosting, and Support Vector Machine), and a deep learning approach (Convolutional Neural Network; CNN) for comparison. We found that the BERT-centric models outperformed all other tested models in terms of all evaluation metrics (accuracy, precision, recall, and F1 score). BioBERT resulted in the highest overall F1 score of 94.49% from the ten-fold cross-validation. Our dataset and coding framework can provide a robust baseline for assessing and comparing new causal sentences extraction techniques. As far as we know, our research breaks new ground by leveraging BERT-centric models for causal sentence classification in the nuclear safety domain and by openly distributing labeled data and code to enable reproducibility in subsequent research.

## 1. Introduction

Extracting causal sentences from event reports, i.e., sentences that mention the cause of an incident, and analyzing them can provide valuable insights into nuclear safety and operational improvements. Therefore, the retrieval of causal sentences is of high practical relevance as it allows for more comprehensive and accurate analyses in Probabilistic Risk Assessment (PRA) [1], Probabilistic Safety Assessment (PSA) [2], and Human Reliability Analysis (HRA) [3]. Databases of unexpected events in nuclear power plants, such as the Licensee Event Report (LER) database maintained and made publicly available by the U.S. Nuclear Regulatory Commission (NRC) [4], usually consist of textual data that are organized in a predefined structure in reports. These reports include detailed descriptions of events during nuclear power plant operations. With the size of databases in the nuclear industry steadily increasing, manual extraction of causal sentences from massive datasets has become challenging for nuclear engineering, safety science, and engineering informatics scholars. This study presents and evaluates state-of-the-art methods for extracting causal sentences from such reports.

The retrieval of causal sentences from nuclear event reports has predominantly relied on time-consuming manual analyses [1], which is unsustainable when considering the increasing volume of databases in the nuclear industry. Several studies have shown that conventional machine learning methods and rule-based approaches can automate the extraction of causal information from nuclear power plant event reports [2, 5, 6]. However, recently developed contextual language models, which can achieve even higher accuracy rates, such as Bidirectional Encoder Representations from Transformers (BERT) [7] and their variants, have not yet been applied to causal sentences identification in the nuclear safety domain.

In this paper, we introduce and evaluate LERCause, an integrated baseline approach for extracting causal sentences from LERs, accompanied by a novel dataset for benchmarking purposes. We build, test, and assess an architecture that integrates BERT-oriented language models, i.e., BERT, BioBERT (a BERT model optimized for biomedical text mining [8]), and SciBERT (a BERT model tailored to scientific literature [9]). We apply this architecture to text data from nuclear power plants to detect causal sentences. We train and validate these models on a corpus of sentences from the abstract section of the LER database. For benchmarking and comparison, we implement one simple heuristic (based on the keywords provided by Zhao et al. [5]), three traditional machine learning methods (Logistic Regression (LR) [10], Support Vector Machine (SVM), and Gradient Boosting (GB) [11]), and a Convolutional Neural Network (CNN) model [12]. To our knowledge, this research pioneers the use of BERT-driven language models for causal sentences extraction from text data from the nuclear safety domain.

The novelty of this study lies in its application of domain-specific, pre-trained language models to the analysis of License Event Reports in the nuclear safety domain. This approach leverages the strengths of SciBERT and BioBERT, which have not been traditionally applied in this context, to enhance the understanding and extraction of technical and safety-related information from LERs. The study also makes the following contributes:

1. Demonstrating the applicability and effectiveness of domain-specific language models in a new and critical area, thereby expanding the potential use cases for these models.
2. Providing a comparative analysis of general and domain-specific BERT-based models, highlighting the importance of domain adaptation for achieving better performance in specialized tasks.
3. Offering insights into the specific challenges and considerations involved in applying NLP techniques to the nuclear safety domain, which can guide future research and practical implementations.

As such, the novelty of this study is reflected in its application of these models to the nuclear safety domain, contributing to both the field of NLP and nuclear safety analysis (https://github.com/jinmok2/LERCause).

This article is structured as follows: Section 2 discusses relevant literature. Section 3 illustrates our dataset, describes the models and methods deployed for causal sentence identification, and explains our experimental design. Section 4 presents our empirical findings. In Section 5, we discuss the implications and limitations of our research. Lastly, Section 6 delivers our concluding thoughts.

## 2. Related work

According to Xu et al., causality indicates a type of relationship between cause and effect, more specifically, that the presence of a cause leads to the occurrence of an effect [57]. Few studies have employed automated methods to discern causality from nuclear power plant event reports. One notable example is the work of Zhao et al., who developed Causal Relationship Identification (CaRI), a rule-based expert system tailored to tease out causal links between events by analyzing abstract sections of LER [5]. From a sample of 70 abstracts, they identified 11 causality-indicative keywords and subsequently formulated 184 rules tied to these keywords to identify causal relationships. When evaluated on a dataset of 330 sentences, their method achieved a 74% recall rate, underscoring the potential usefulness of rule-based systems in automatically detecting causal relationships f textual content.

As SVMs have shown promising performance for text classification [13, 14], several researchers have used SVMs to extract causality indicating content from free-text reports. For example, Pence et al. developed an SVM classifier to analyze the organizational causal factors of accidents from nuclear power plant event reports [1]. They trained an SVM on more than 6,000 abstracts and the Cause sections of LERs using features based on entropy rankings. The results of their experiment on 200 annotated LERs as test data produced a perfect recall of 1.00 at the cost of a lower precision of 0.672. In a follow-up study, Yang et al. applied SVMs to three different datasets from LERs (i.e., abstract, Causal section, and a mixture of abstract and Causal section), reporting about 80% F1 scores [6]. Their experiments showed that increasing the training dataset size can improve SVM-based classification accuracy.

Studies outside the nuclear engineering and safety domain have extensively used machine learning techniques to extract event causality from textual data [13–16]. For example, Zhao’s team developed a Restricted Hidden Naive Bayes (RHNB) classifier to retrieve causal relationships by considering the interactivity between causal connections and lexical-syntactical features [16]. They trained an RHNB classifier on 2,682 sentences from the SemEval-2010-Task8 corpus, a widely used dataset for semantic relationship classification [17], and reported a recall of 0.841 and precision of 0.873.

Other researchers have used deep learning approaches and demonstrated better performance than conventional machine learning methods for causal relations identification [18–21]. For example, Li et al. introduced Knowledge-oriented CNN (K-CNN) for causal relationship identification. They showed that adding prior lexical knowledge from external linguistic resources into a neural network model can improve causal relation identification performance [19]. Their model resulted in a recall of 0.909 and precision of 0.946 and showed that deep learning can outperform traditional machine learning methods like SVMs in causality detection. In a recent study, Li et al. proposed a model named SCITE for causality extraction based on BiLSTM-CRF (Bidirectional Long Short-Term Memory and Conditional Random Field) with transferred contextual string embeddings [21]. By reporting a recall of 0.860 and precision of 0.849, they showed that their BiLSTM-based models produced better results than CNN-based models in their experiments.

Pre-trained language models, including BERT and its variants, have recently demonstrated outstanding performance in many natural language processing (NLP) tasks [22, 23]. It has also been reported that language models created from unsupervised pre-training can improve the performance of causality extraction across different text datasets and domains, including biomedical literature [24], clinical notes [25], and news articles [26]. However, these contextual language models have not been utilized for causality extraction tasks in the nuclear safety domain. This study examines the usage of pre-trained language models for extracting causal sentences from nuclear power plant event reports. Table 1 summarizes previous research on causal content extraction; highlighting models and datasets.

**Table 1:** Overview of prior work on causal content extraction from text datasets (A = Accuracy, R = Recall, P = Precision, F = F1 score).

Citation(Year)	Domain	Corpus	Data Size & Type	Model	Target Class	Performance(Max)
Zhao et al. [5] (2019)	Nuclear	LER	70 Abstracts,	Rule-based	Causal relation	R 0.740
			330 Sentences			
Pence et al. [1] (2020)	Nuclear	LER	6,165 Abstracts 6,067 Cause sections	SVM	Organizational causal LER	A 0.890
						P 1.000
						R 0.672
Zhao et al. [16] (2016)	General	SemEval-2010-Task8	2,682 Sentences	RHNB	Causal relation	P 0.873
						R 0.841
						F 0.856
Li et al. [19] (2019)	General	SemEval-2010-Task8	11,647 Sentences	K-CNN	Causal relation	P 0.946
		Causal-TimeBank				R 0.909
		Event StoryLine				F 0.926
Li et al. [21] (2021)	General	SemEval-2010-Task8	5,254 Sentences	CNN	Causal relation	P 0.849
				LSTM		R 0.860
				SCITE		F 0.848
Reklos et al. [24] (2022)	Biomedical	Bio-causal	5,061 Sentences	BERT	Causal sentence	F 0.881
		DCLS		SciBERT		
				BioBERT		
Khetan et al. [25] (2022)	Clinical	2018 n2c2	2,714 Sentences	BERT	Causal relation	F 0.560

## 3. Materials and methods

### 3.1 Dataset

**Fig 1:**
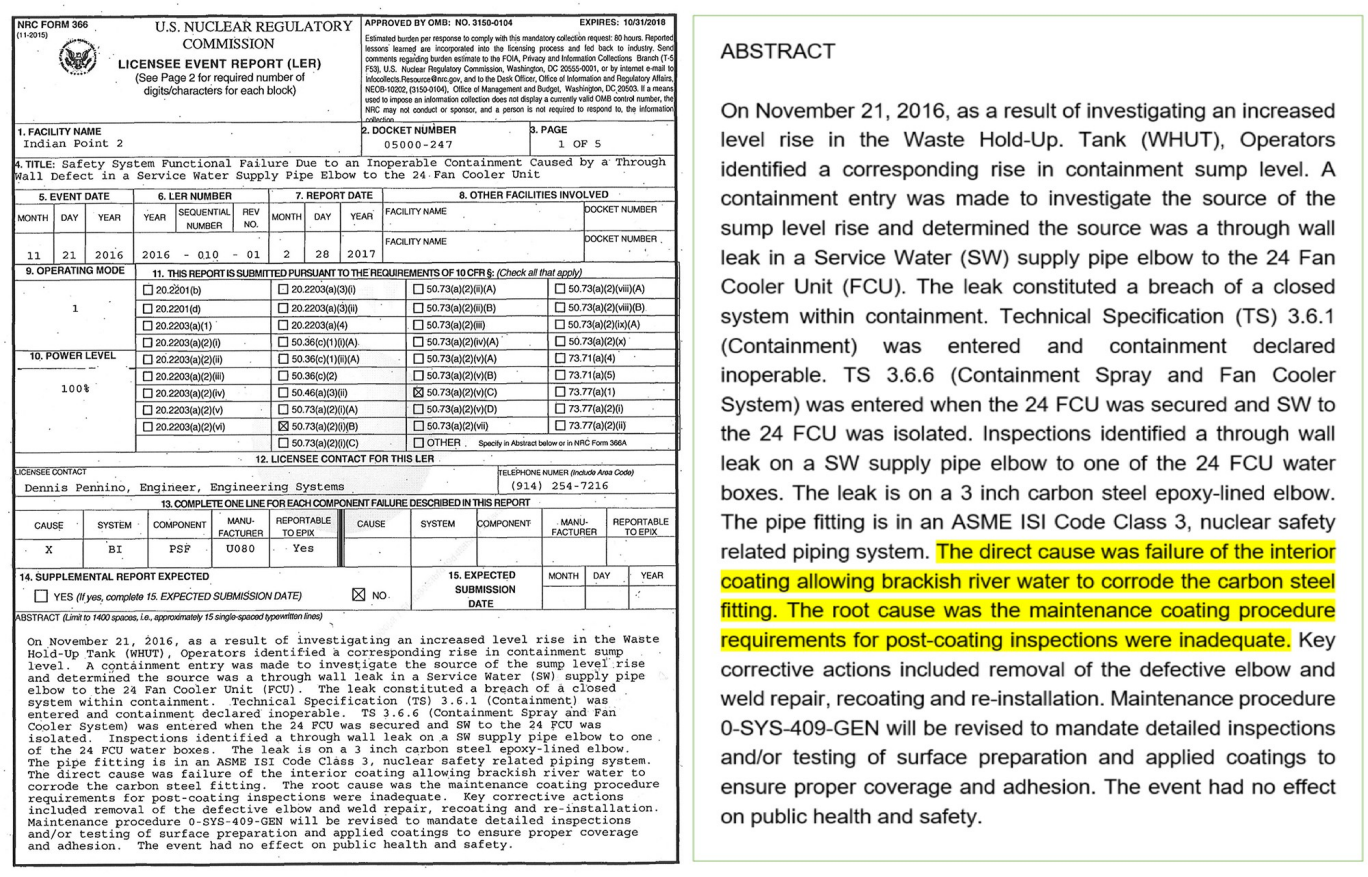
LER sample (left) and Causal sentences (right, highlighted).

Our dataset consists of sentences derived from abstracts of texts from the Licensee Event Report (LER) corpus. The LER database is an open source of data reporting unexpected plant events caused by human errors and equipment failure from 1980 to the present (https://lersearch.inl.gov/LERSearchCriteria.aspx). Nuclear power plants must submit LERs to the Nuclear Regulatory Commission (NRC) when “reportable events,” such as a plant shutdown, radioactive release, and other events that can impact plant safety occur [27]. LERs are semi-structured documents with various data fields, such as a facility’s name, report date, and event date. These reports include checkboxes for data input and free-text fields. Users can input an abstract summarizing the event and a detailed narrative within these fields. The narrative section includes an overview on a given event, insights from technical investigations, causal factors, corrective measures taken, and possibly parallels to past events of a similar nature. The abstract mainly presents an event summary and causal sentences. The latter are the target of our causal sentences prediction task (see Fig 1).

With standardized data and comprehensive information provided in free text, LER can provide insights into nuclear safety, plant operational management, and regulatory decision-making. For instance, automatic analysis of human errors using LER can offer a guideline for evaluating and modifying maintenance rules to screen functional failures and prevent defect-causal actions [28]. Such potential advantages of LER have facilitated non-machine learning studies in many research areas, including Probabilistic Risk Assessment [1, 2, 6], Human Performance Analysis [29], Multi-Unit Dependency Analysis [30], and Industry Trends Programs [31]. However, most studies have been based on coded and structured data from LER [1]. In addition, a limited number of studies using LER’s free-text [1, 6, 32, 33] has applied machine learning and other NLP techniques at the paragraph, section, or document level except for [2, 5]. Given this context, our study is the first attempt to apply deep learning techniques to causal sentences prediction based on a sentence-level LER dataset.

There are several challenges to be addressed when handling textual data in nuclear science. First, textual data in the nuclear science domain, such as License Event Reports (LERs), often contain highly technical and specialized language. This complexity poses a significant challenge for NLP models, which must accurately interpret and classify these terms. This necessitates the use of models like SciBERT and BioBERT, which are pre-trained on scientific and biomedical literature, respectively, to also capture domain-specific nuances. Second, sentences in LERs can be context-dependent, with causal relationships often being implied rather than explicitly stated. This requires models to not only understand individual sentences but also the broader context in which they appear; making the task more complex than simple sentence classification. Finally, LERs can vary significantly in length, structure, and detail, necessitating a flexible approach that can handle diverse document formats and sizes while maintaining high classification accuracy.

Among the LERs from 2000 to 2019, 1,200 abstracts were randomly selected, which led to a total number of 10,608 sentences. Two human researchers annotated all sentences in the set. Manual annotation refers to the meticulous and human-driven process of assigning labels to specific data points for machine-learning classification tasks [34]. If a sentence expresses the causality of an event, it was labeled as 1 (Causal); otherwise, it was labeled as 0 (non-Causal). The two annotators then reviewed the sets individually and met to discuss any disagreements. The inter-rater reliability (see Table 2) among these two annotators was measured using Cohen’s kappa [35]. This kappa statistic (κ) can be obtained by the Eq (1):

$$\kappa =\frac{P\left(r\right)-P\left(h\right)}{1\;-P\left(h\right)}$$ (1)

There, *P(r)* is the actual observed agreement, and *P(h)* is the hypothetical probability of random agreement. If every annotation from each annotator agrees, then κ = 1. The measured kappa score was 0.973, which implies almost perfect agreement [36]. Finally, the labeled dataset, consisting of 2,096 Causal and 8,512 non-Causal sentences, was separated into ten folds (n = approximately 1,061 sentences per fold) using a consistent random seed for the ten-fold cross-validation.

**Table 2:** Inter-rater reliability analysis for annotators on LER sentences.

		Annotator 2	
		Causal	non-Causal
Annotator 1	Causal	2,096	50
	non-Causal	41	8,421

### 3.2 Experiments

**Fig 2:**
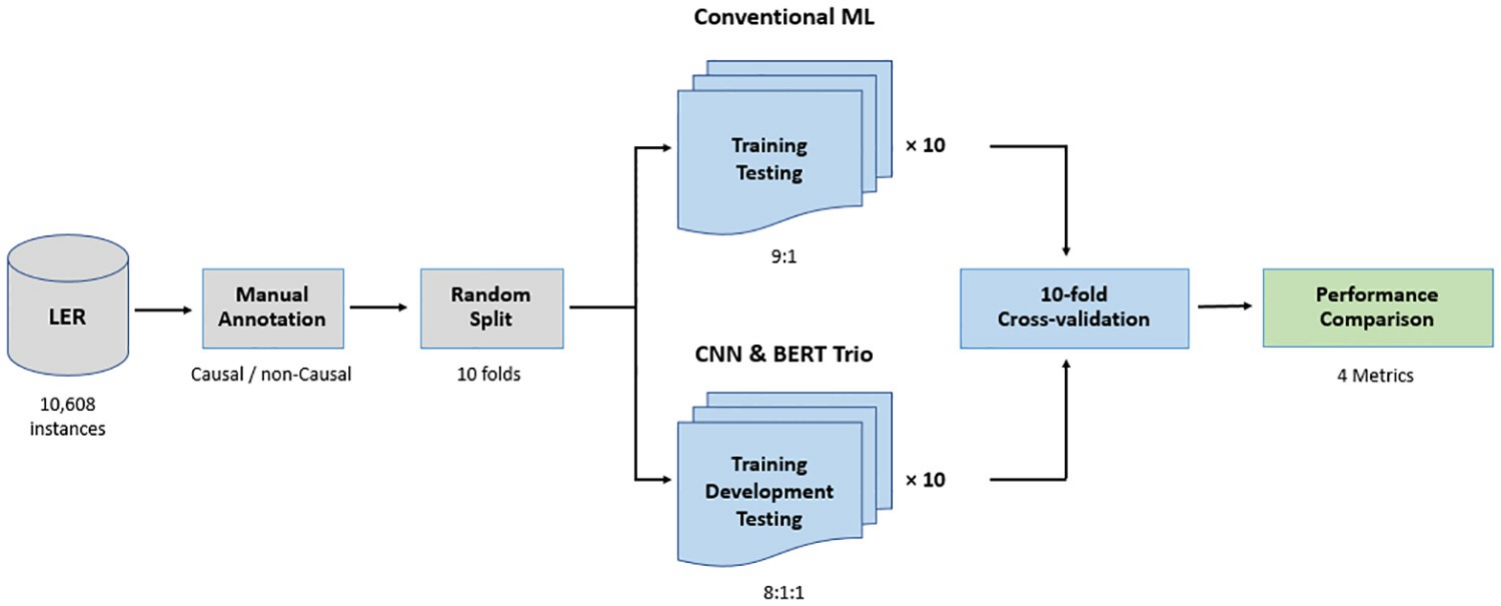
Overview of causal sentence classification methodology.

Extracting causal sentences from LERs is a binary classification task. In this study, we employed three traditional machine learning classifiers (LR, GB, and SVM) and a deep learning approach (CNN) based on their prior efficacy in numerous classification tasks [12, 37, 38]. We applied BERT-based techniques as potential game-changers, utilizing three specific models: BERT, BioBERT, and SciBERT. Additionally, we applied a basic heuristic based on keywords to benchmark against the aforementioned algorithmic methods. All models except for the heuristic method were evaluated based on the ten-fold cross-validation. Each traditional machine learning method was trained on nine folds and tested on a fold through each run. To evaluate CNN and BERT-centric models, eight folds, a fold, and a fold were assigned through each run for training, development, and testing, respectively. The development set was used for tuning the CNN and BERT-centric model to identify optimal configurations through each run. The heuristic model was tested on each fold. We reported each classifier’s average performance scores. Fig 2 provides an overview of the causal sentence classification process.

#### 3.2.1 BERT-based models

*3*.*2*.*1*.*1 BERT*. BERT is a model that generates contextualized word embeddings using a bidirectional approach to the Transformer architecture, allowing it to consider a word’s context [39]. BERT is pre-trained on large corpora from Wikipedia and the Books Corpus. In this section, we briefly explain the operating structure of BERT in three stages: input representation, pre-training, and fine-tuning (see [7] for a more detailed description of BERT). To generate input tokens from corpora, BERT uses a word-piece tokenization method that splits each word into sub-word units called WordPieces [40]. The set of tokens includes two special tokens: classification token *[CLS]*, added at the starting point of each input sequence, and sentence separation token *[SEP]*. Then, position embedding, segment embedding, and token embedding are summed to produce input representation for every token.

BERT’s pre-training process comprises two unique training tasks: Masked Language Model (MLM) and Next Sentence Prediction (NSP). Before BERT was introduced, language models used a right-to-left and/ or left-to-right training approach to predict following words [22], or tried a combination of one-way models [41]. Unlike these language models, BERT achieved left and right (i.e., bidirectional) pre-training using the MLM procedure. In MLM, input tokens are randomly masked and replaced with the *[MASK]* tokens. Predictions are made for those masked tokens by using the masked tokens’ left and right context with a transformer. This neural network architecture learns this context using a multi-head attention mechanism. Next, in the NSP task, BERT can be pre-trained to predict whether a pair of sentences have a relationship. For instance, NSP checks whether sentences A and B from the pre-training example are adjacent to each other and gives them one of the labels of IsNext or NotNext. This NSP-based pre-training process is especially beneficial for sentence pair tasks such as natural language inference and question answering [42].

**Fig 3:**
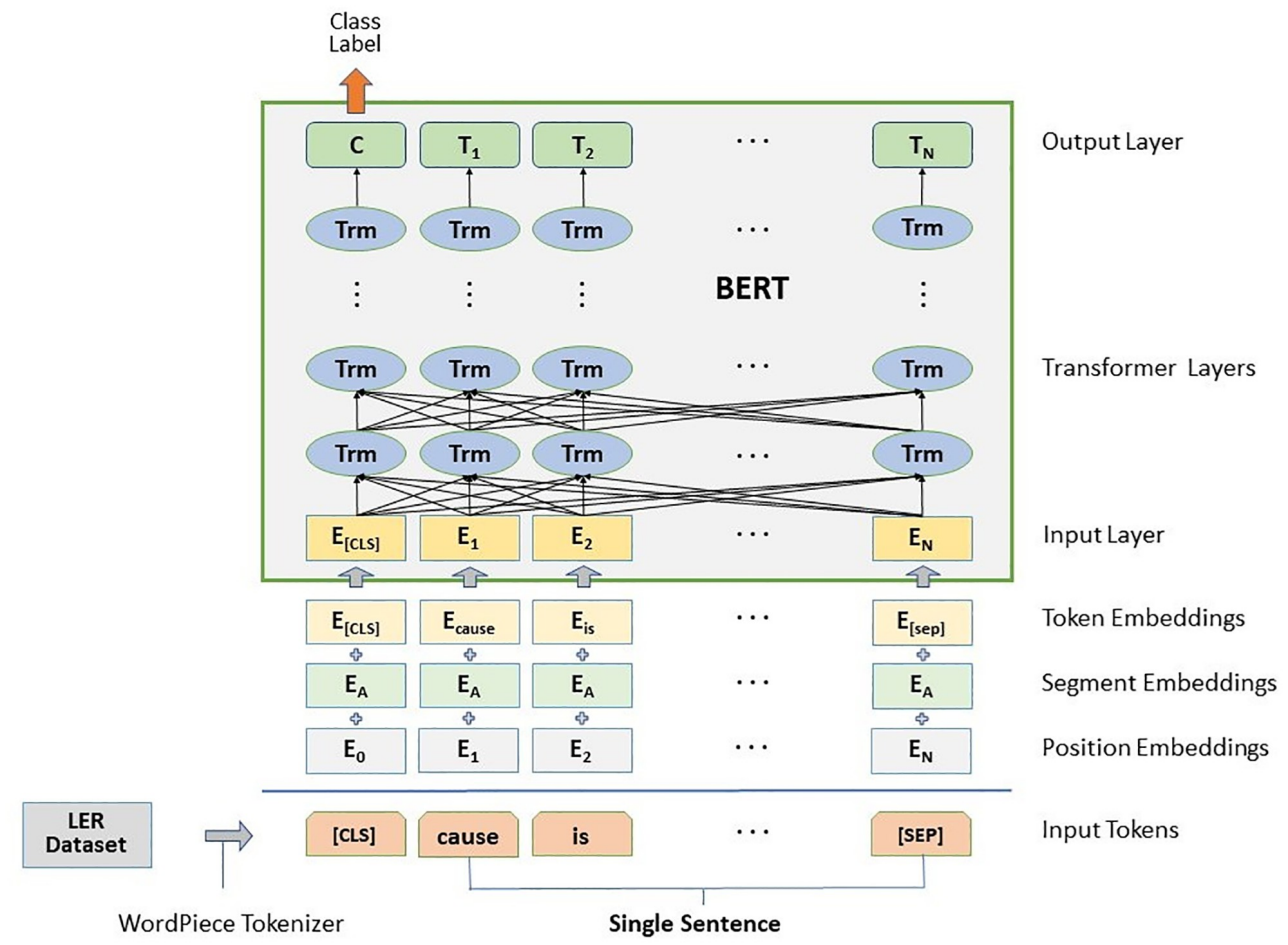
Illustration of BERT architecture on causal sentence classification (see [7] for more details).

We can fine-tune BERT using small datasets for a specific NLP task (e.g., causal sentence prediction). A tailored output layer can be connected to the pre-trained BERT with minimal parameter tuning. Also, many variants of BERT have already been pre-trained on domain-specific corpora, and researchers can access some of them [8, 9, 43, 44]. In this study, we try the pre-trained weights of the BERT base and a cased version: bert-base-case. Fig 3 illustrates the architecture of BERT on sentence classification tasks.

*3*.*2*.*1*.*2 BioBERT*. BioBERT is a BERT-oriented model for the biomedical domain [8]. Besides the original BERT’s corpora, BioBERT was pre-trained on extensive biomedical data from PubMed (https://pubmed.ncbi.nlm.nih.gov/) and PubMed Central (PMC) (https://www.ncbi.nlm.nih.gov/pmc/). PubMed is an open database of biomedical abstracts and citations, and PMC is a digital full-text archive of biomedical articles. BioBERT has shown high performance in medical entity recognition, biomedical relation identification, and Randomized Control Trials (RCTs) classification [45]. BioBERT is freely available for download, and the source code is open for fine-tuning. The authors of BioBERT have experimented with different combinations of these corpora and have released different versions of the BioBERT model based on the data and the number of pre-training steps. For instance, this study uses one of these versions—BioBERT-Base v1.1.

*3*.*2*.*1*.*3 SciBERT*. SciBERT is based on the architecture of BERT but pre-trained on 1.14M scientific papers from the computer science (18%) and biomedical (82%) domains from Semantic Scholar (https://www.semanticscholar.org/) [9]. The average paper was reported to have 154 sentences, amounting to 2,769 tokens, cumulatively giving a corpus of 3.17B tokens. SciBERT has been employed for various NLP tasks in the scientific domain, including sentence classification, sequence labeling, and question answering [9]. It has shown superior performance over BERT and produced outstanding results for several NLP tasks [9, 24, 45]. For this study, we utilize the ’scibert-scivocab-cased’ version.

#### 3.2.2 Pre-processing

We leveraged various BERT-based models to compare their accuracy to conventional machine learning methods, where removing extraneous text is pivotal for feature selection. In contrast, language models like BERT and its derivatives do not have this need because they utilize complete word sequences—including punctuation and stop words—to capitalize on the sentences’ semantic and contextual characteristics [7–9]. As a result, the following preprocessing steps were exclusively tailored for the traditional machine learning techniques we used (LR, SVM, and GB): (1) All words were converted to lowercase. (2) Sentences were tokenized using Python’s Natural Language ToolKit (NLTK) [46]. (3) Punctuation and stop words were removed. (4) For dimensionality reduction, words were stemmed with the Porter Stemmer available in NLTK.

After pre-processing, Term Frequency Inverse Document Frequency (TF-IDF) [47] was calculated as a token-level feature for the conventional machine learning classifiers. Computed at the sentence level, a TF-IDF statistic indicates the relative frequency of each word in a sentence contrasted to the inverse frequency of that word over all sentences in a given corpus. Words that frequently appear in smaller number of sentences tend to have higher TF-IDF scores than commonly appearing words. TF-IDF (*v*) was calculated as per Eq (2):

$$v_{\text{w},\text{s}}=f_{\text{w},\text{s}}*\text{log}\left(\left|S\right|/f_{s,S}\right)$$ (2)

There, *S* is the sentence corpus, *w* is a word, *s* is an individual sentence (*s* є *S*), *f*_w, s_ is the frequency at which *w* appears in *s*, |*S*| is the corpus size, and *f*_*s*, *S*_ is the number of sentences in which *w* emerges in *S* [48]. Using the BERT-based models, we do not need pre-processing procedures except tokenization. Unlike BERT’s workpiece tokenizer, the tokenization method of the BERT variants may vary depending on the texts on which the model was pre-trained. In this study, tokenizers were loaded from the Hugging Face transformers library (https://huggingface.co/) for the BERT-centric models [49].

#### 3.2.3 Implementation background

Our experiments were conducted on SageMaker, a cloud-based Amazon Web Services (AWS) platform that facilitates building, training, and deploying machine learning methods. For the classic machine learning classifiers, we used the ‘ml.p3.2xlarge’ notebook instance. These classifiers were implemented from Scikit-learn (version 1.0.2), a widely-used Python library for machine learning [50]. Default parameters were used to train these conventional machine learning methods. For example, the Logistic Regression (LR) model utilized L2 regularization and was constrained to 100 iterations for convergence. The Gradient Boosting (GB) algorithm was trained with 100 estimators (trees) and a 0.1 learning rate.

We employed Keras (version 2.13.1) and TensorFlow (version 2.13.0) for our CNN model (https://www.tensorflow.org/). Keras is a Python-driven machine learning API built atop the TensorFlow deep learning framework [51]. We utilized a ’ml.p3.2xlarge’ notebook with an NVIDIA Tesla V100-SXM2-16GB GPU. During training iteration, GPU utilization peaked at 15.6 GB of memory. The CNN architecture began with a word embedding layer constructed from the training dataset to encapsulate semantic relations. Subsequent tensors transitioned via twofold convolutional layers, sequentially adopting two and three kernel sizes. Every layer integrated 512 filters and leveraged ReLU for its activation function. Outputs underwent a max pooling operation, and the culminated max-pooled scores were combined into one layer. We incorporated a 0.5 dropout rate for overfitting mitigation. The fully connected output layer used a sigmoid function to generate a probability that a result belongs to a class (0 or 1). In congruence with the BERT-based models’ environment, batch size was set to 16 with a 4-epoch training loop. We selected the binary cross-entropy for the loss function and ADAM [52] for the optimizer. The input’s maximum length for the embedding layer was empirically determined at 150 based on the dataset’s word distribution.

To fine-tune BERT-based models for training, we used the Hugging Face transformers library (version 4.30.0) (https://github.com/huggingface/pytorch-transformers) in conjunction with the PyTorch framework (version 2.0.1) (https://pytorch.org/). This library offers a robust API with pre-trained models for diverse NLP tasks. PyTorch, an open-source tensor library from Facebook, is a prominent deep-learning platform. For each training and testing phase on SageMaker, we employed an ’ml.p3.2xlarge’ notebook backed by an NVIDIA Tesla V100-SXM2-16GB GPU. GPU memory usage reached its 15.6GB capacity for training iteration. Adopting guidelines from [7], we used a 2e-5 learning rate and executed a training loop over four epochs with a batch size of 16. The token length ceiling was fixed at 150, mirroring the CNN model’s embedding layer input length. The binary cross-entropy was designated as the loss function, while ADAM achieved optimization. After each optimization cycle, the model’s performance was assessed against our development fold.

#### 3.2.4 Heuristic method

We implemented a straightforward keyword-matching approach using keywords supplied by Zhao et al. [5], basically a heuristic method, as a benchmark for the other methods. After removing punctuation, sentences were scanned for a given set of keywords. If a sentence contained at least one keyword or key phrase from the set, it was labeled as 1 (Causal), otherwise as 0 (non-Causal). The keywords utilized for this heuristic strategy can be found in Table 3.

**Table 3:** Keywords list for the heuristic method.

**Keyword**	‘result in’, ‘caused’, ‘due to’, ‘be caused by’, ‘result from’, ‘as a result of’, ‘cause of’,
	‘causes of’, ‘in response to’, ‘because’, ‘because of’, ‘attributed to’, ‘lead to’

## 4. Results

### 4.1 Evaluation metrics

In this section, we present the evaluation outcomes of our models for predicting causal sentences. Four commonly used metrics from information extraction were used for analysis: accuracy, precision, recall, and F1 score. To define these four metrics, the following components are considered:

- TP (True Positives): Instances belonging to and classified as the target class (Causal).
- FP (False Positives): Instances misclassified as the target class (Causal) that do not belong to it.
- FN (False Negatives): Instances that belong to the target class (Causal) but are classified as non-Causal.
- TN (True Negatives): Instances classified as non-Causal that don’t belong to the target class (Causal).

Using these components, accuracy, precision, recall, and F1 can be defined as:

- Accuracy (= TP + TN / (TP + FP + FN + TN)): This indicates the ratio of correctly predicted sentences to all the sentences assessed.
- Precision (= TP / (TP + FP)): Of the sentences a model predicts as belonging to the Causal class, this metric represents the fraction that was correctly identified.
- Recall (= TP / (TP + FN)): Out of all the genuine causal sentences, this denotes the fraction the model correctly identified.
- F1 Score (= (2* Precision*Recall) / (Precision + Recall)): A metric representing the harmonic mean of precision and recall.

### 4.2 Impact of classification algorithm on model performance

Table 4 shows the evaluation results for the heuristic method, three traditional machine learning classifiers, CNN, and BERT-centric models. The heuristic approach is outperformed by all other methods on all metrics. The straightforward keyword-matching was comparatively unsuccessful, e.g., in cases where the target words were absent or occurred in a spelling variation (e.g., ‘led to’), leading to a recall score of 71.52%. Among the traditional machine learning methods, GB performed better than LR and SVM in terms of accuracy, recall, and F1. LR had the highest precision among all the traditional machine learning methods. While the traditional machine learning methods achieved precision rates of over 90%, their recall was considerably lower (73%–83%). CNN performed better than the conventional machine learning methods but worse than the BERT-based models in terms of accuracy, recall, and F1. Its precision was superior only to the heuristic model.

**Table 4:** Evaluation summary for all models from the ten-fold cross-validation. Values in bold represent the top performance per metric across all models.

Method	Model	Accuracy (%)	Precision (%)	Recall (%)	F1 (%)
Heuristic	Keywords	90.08	76.71	71.52	74.01
Conventional Machine Learning	LR	93.70	93.71	73.05	82.08
	SVM	95.17	92.70	82.02	86.99
	GB	95.24	91.91	83.25	87.34
Neural Networks	CNN	96.51	91.62	90.75	91.13
BERT-based	BERT	97.63	95.44	92.46	93.92
	BioBERT	**97.85**	**95.72**	93.32	**94.49**
	SciBERT	97.40	93.45	**93.51**	93.44

CNN and the BERT-based trio also achieved precision rates of 90% but also higher overall F1 scores due to improvements in recall. Even the BERT-based model with the lowest performance exceeded the conventional machine learning methods and CNN across all metrics except precision. Among all assessed models, BioBERT did best with an accuracy of 97.85%, a precision of 95.72%, and an F1 rate of 94.49%. SciBERT had the highest recall rate of 93.51%. BERT did slightly better than SciBERT and slightly worse than BioBERT, indicating that domain specific model training can improve prediction performance but does not always do so. Overall, BERT-based models consistently surpassing traditional machine learning methods and CNN in performance.

### 4.3 Impact of dataset size on model performance

We varied the training dataset size to see how sample size impacts model performance. The labeled dataset was separated into training, development, and testing subsets, comprising 80% (8,486 sentences), 10% (1,061 sentences), and 10% (1,061 sentences) of the total data. Then, we randomly sampled sentences using a consistent random seed from the training subset, were with an increment ratio of 0.1 (i.e., 849, 1,698, …, 8,486). We only changed the training data size while keeping all the other settings unchanged, such as optimization, development and testing dataset, and hyperparameters. This allows us to isolate the impact of training dataset size on model performance. As a result, we ran the traditional machine learning classifiers and BERT-based models on ten different sizes (0.1 to 1.0) of data. To ensure consistency and offset the innate variability of classifiers, we replicated this experimental procedure (either training-testing or training-development-testing) for each algorithm ten times.

**Fig 4:**
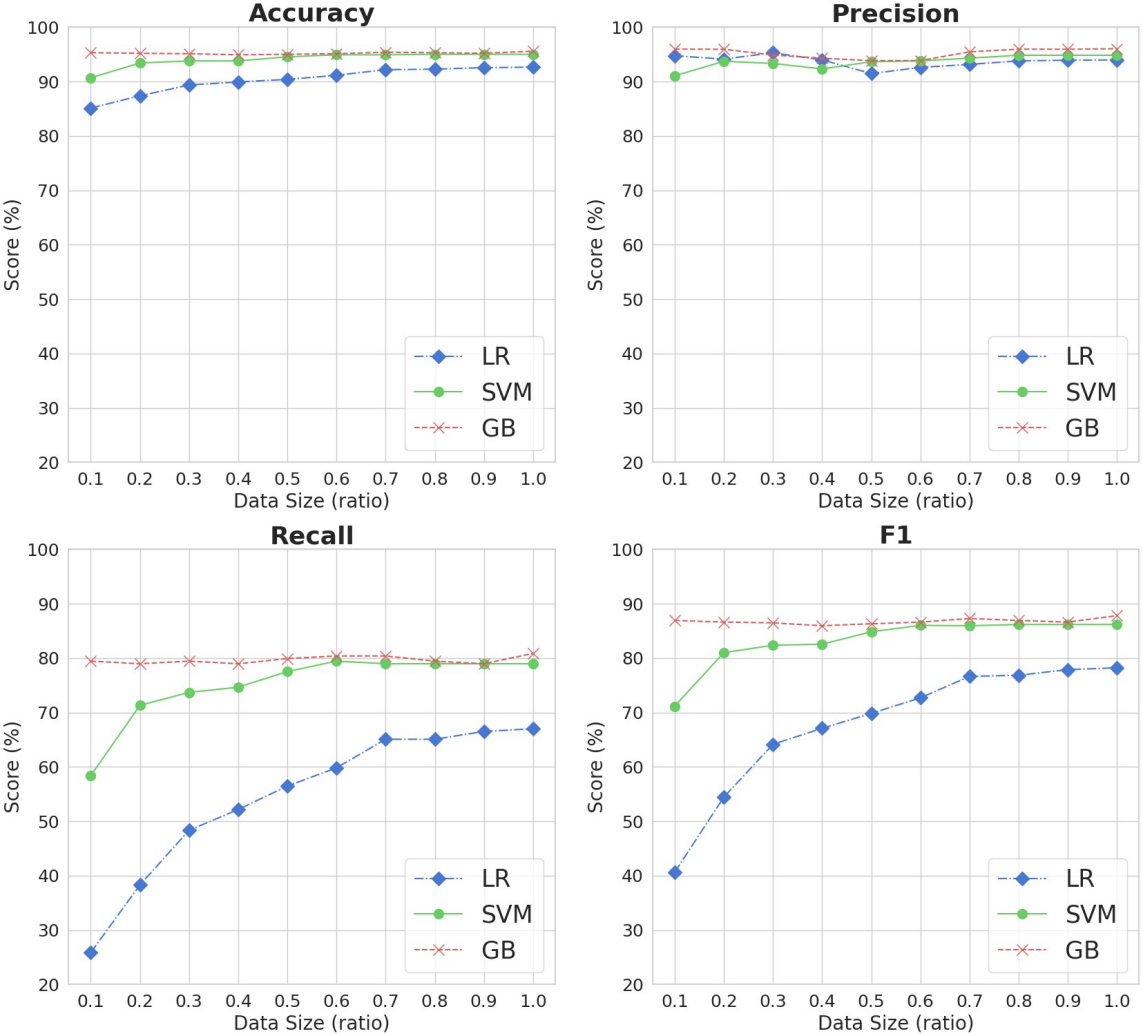
Evaluation results of classical machine learning methods based on dataset size.

**Fig 5:**
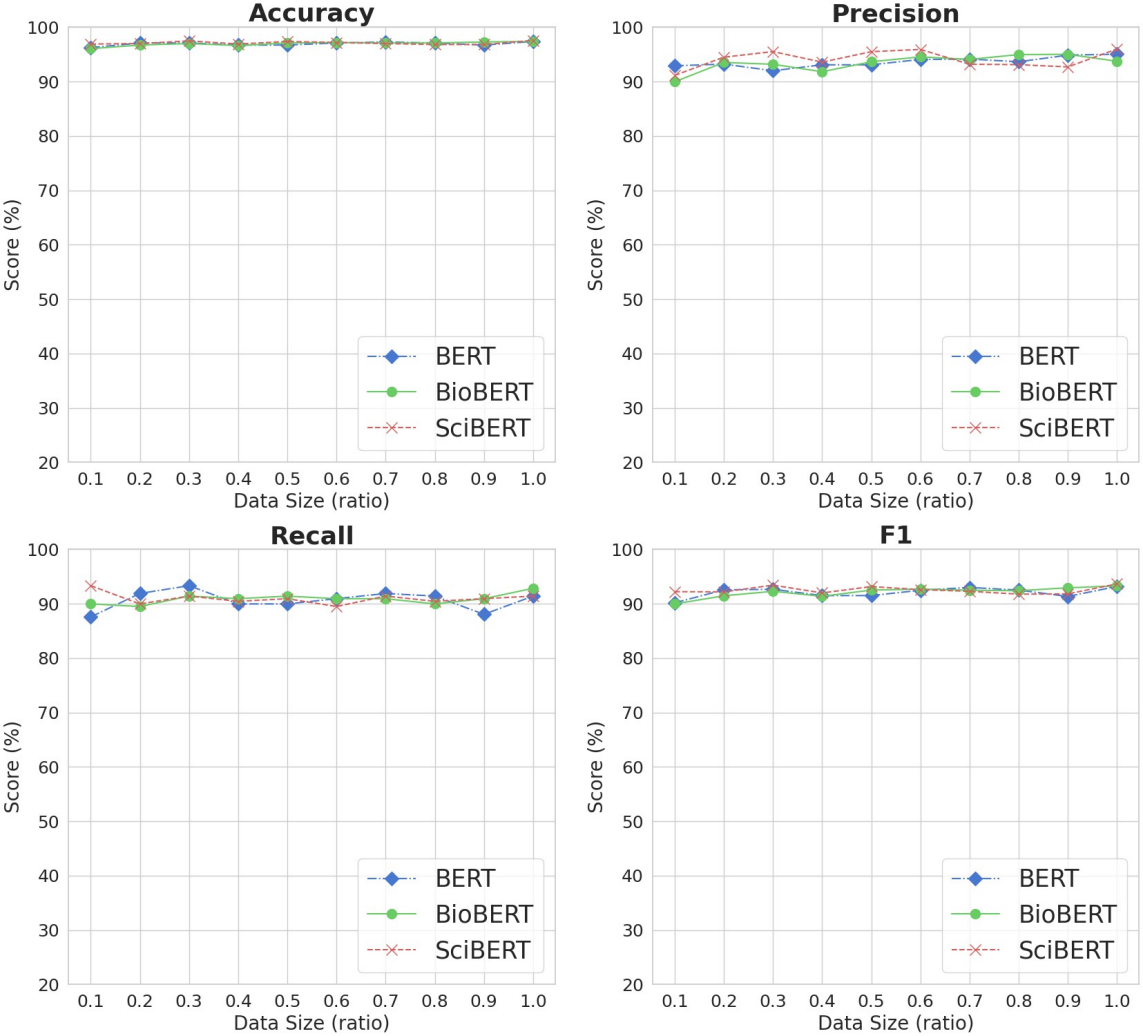
Evaluation results of BERT-based models based on dataset size.

Figs 4 and 5 show the average results of ten iterations for traditional machine learning and BERT-based classifiers, respectively, based on training dataset size for each evaluation metric. We found that dataset size affects recall and thereby the F1 metric. For example, the LR model showed a rapid growth curve with respect to recall scores, indicating that the model performs better with more data. In contrast, we saw only a slight change in precision for all traditional machine learning classifiers. The GB model was not affected by dataset size for any evaluation metric, implying that the model does as well on big as on small data.

Similarly, the training dataset size does not impact the performance of BERT-based models. As illustrated in Fig 5, all three BERT models had consistent outcomes irrespective of sample size across all evaluation metrics. Moreover, the BERT-based models exhibited steadier recall scores than the traditional machine learning methods, which deteriorated with smaller sample sizes. This finding underscores the ability of BERT-based models to perform well on smaller datasets while effectively maintaining stable performance.

### 4.4 Error analysis

In this study, the possible types of errors are called false negatives (FN) and false positives (FP). False negatives represent instances that belong to the Causal class but are misclassified as non-Causal, while false positives refer to non-Causal sentences wrongly classified as belonging to the Causal class. In this error analysis, we reported the average scores of FN and FP for each classifier. Our qualitative inspection of these errors showed that false negatives occurred when the cause of the accident was not explicitly specified in the sentence or had to be inferred through the context of other sentences. On the other hand, classifiers caused false positives when a sentence did not express an event’s causality but included clues related to the cause of the accident (e.g., ‘cause’, ‘result in’, and ‘failure’). Examples of these errors are shown in Table 5.

**Table 5:** Examples of errors (FN = False Negatives, FP = False Positives).

Type	Examples
**FN**	*“The maintenance history revealed that the bearing was incorrectly installed at the vendor’s facility during the last pump overhaul in late August 2003*.*”*
	*“Detailed requirements were not provided to Power Control*, *and NMP1 Design Engineering failed to verify that Power Control could maintain sufficient Line 4 voltage with Line 1 out of service*.*”*
**FP**	*“The cause of the inadequate lubrication is continuing to be pursued*.*”*
	*“During the interval that these orifices were installed*, *there was no plant event which resulted in an actual failure of the AFW system*.*”*

In the nuclear safety domain, false negatives can pose higher risks that false positives since false negatives let potential causal factor of an accident go undetected, introducing a false sense of safety. A false positive means a false alarm while a false negative is a broken alarm. As shown in Table 6, Bert-based models reported fewer false negatives than the other classifiers. SciBERT showed the least false negatives (FN = 13.6) among all models. All models produced more false negatives than false positives except for SciBERT. It is necessary to improve the models to reduce false negatives in future studies.

**Table 6:** Summary of errors (TP = True Positives, FP = False Positives, FN = False Negatives, TN = True Negatives).

Method	Model	TP	FP	FN	TN
Heuristic	Keywords	149.9	45.5	59.7	805.7
Conventional Machine Learning	LR	153.1	10.3	56.5	840.9
	SVM	171.9	13.6	37.7	837.6
	GB	174.5	15.4	35.1	835.8
Neural Networks	CNN	190.2	17.6	19.4	833.6
BERT-based	BERT	193.8	9.3	15.8	841.9
	BioBERT	195.6	8.8	14.0	842.4
	SciBERT	196.0	14.0	13.6	837.2

## 5. Discussion

Our main finding is that the considered BERT-based models outperform the tested heuristic method, conventional machine learning methods, and CNN method when telling apart causal and non-causal sentences in the given domain and data. BioBERT performed best among all examined models, with an F1 score of 94.49%.

A possible explanation for the comparatively high performance of BERT-based models is their foundation on extensive pre-training. For instance, the BERT variants we utilized were trained on the expansive Books Corpus, spanning over 800 million words. When repurposed for our study, such extensively pre-trained language models seem to be able to consider context and nuances in the LER data. Traditional word embeddings tend to generate uniform vectors for words, irrespective of their use cases. In contrast, BERT-based models use context-sensitive vectors [7]. This enables them to capture different word contexts and meanings, which likely empowered the BERT models to detect truly causal sentences and enhance recall, which seemed harder for the conventional machine learning algorithms.

Besides the mentioned performance advantages, BERT-based models offer procedural simplicity for causal sentence classification: they bypass the pre-processing and feature selection phase which both are indispensable for traditional machine learning methods. One of the practical implications of our experiments is that BERT-based models can deliver stable results even with small data, which can be essential in specialized application cases. Such model resilience to training dataset size can reduce the labor and time invested in data annotation. Furthermore, our methodology allows researchers to refine our models by generating classification probability scores per sentence. Sentences that hover below a defined confidence threshold could then be earmarked for manual classification.

While there are numerous other popular models, such as GPT-3 [22], RoBERTa [53], and T5 [54], the selection of three BERT-based models can be justified by their specific training regimes and demonstrated efficacy in handling domain-specific texts. Specifically, SciBERT and BioBERT, which are pre-trained on scientific and biomedical literature, are well-suited for handling the technical and specialized language prevalent in nuclear safety reports. On the other hand, GPT-3, although powerful, is computationally intensive and more generalized, lacking the domain-specific pre-training of SciBERT and BioBERT. RoBERTa, an optimized version of BERT, has shown high performance on NLP tasks [53] but does not offer the same specialized training as SciBERT or BioBERT. In addition, T5, which excels in sequence-to-sequence tasks, may not have been as effective for the classification tasks addressed in this study as the other tested BERT-based models.

The extraction of text elements indicating causal events has been extensively studied in NLP [55–59], including by using language models such as Causal BERT [60]. Building upon prior work, this study focuses on classifying causal sentences using BERT-oriented models, presenting several advantages over alternative methods. First, this approach eliminates the initial step of processing large-scale textual data, allowing lower (computational) costs. In contrast, direct causality extraction often requires complex and resource-intensive algorithms, which can be challenging to implement and maintain [56, 57]. In addition, the classification approach used in this study enables a stepwise analysis that can be incrementally improved. This modularity allows researchers to build upon the initial classification, adding more sophisticated causality extraction techniques in later stages if needed. Finally, the classification output is interpretable for practitioners in the nuclear safety domain and can be easily integrated with other NLP techniques, such as dependency parsing and knowledge graph construction, which can further enrich the analysis of causal sentences.

A significant limitation of our research is that we worked with one specific dataset in the proposed architecture. Looking ahead, we aim to broaden our research scope by transitioning from abstracts to the full texts of LER. Additionally, we plan to harness automated techniques for datasets beyond LER (e.g., the Institute of Nuclear Power Operation (INPO) database [61]). Another constraint of our methodology is its focus on extracting explicit causal sentences. In the context of nuclear safety, missing implicit causal sentences can lead to significant oversight in understanding the root causes of incidents, potentially compromising safety measures. To mitigate false negatives, combining multiple models, each specializing in different aspects of causal inference (e.g., explicit markers, context-based inference), might improve overall accuracy and reduce blind spots. Another strategy would be to implement post-processing techniques to re-evaluate sentences flagged as non-Causal to help catch implicit causal sentences that might have been initially missed.

Also, in event reports, causality often extends across multiple sentences and can even span an entire document [55]. For addressing inter-sentence causality, extending the context window used by the model to include multiple sentences or even entire paragraphs might help to capture long-ranging causal expressions. Such an approach would leverage the transformer architecture’s capability to handle long-range dependencies. Future studies also could utilize graph-based models, such as Graph Convolutional Networks (GCNs) for document-level causality extraction [62–64]. For example, Zeng et al. introduced a combination system of GCN for relations extraction within an entire document [62]. A final constraint of our study is our exclusive experimentation with three pre-trained models, while not using other existing approaches like Causal BERT [60] or GPT-4 [65] which would need to be done to find out if BERT-based models generally outperform traditional methods and other state-of-the-art approaches for classifying causal sentences in data from the nuclear safety domain. We expect our study to support future work using various deep-learning techniques for causal sentences extraction.

## 6. Conclusion

Correct causal sentence identification in the nuclear safety domain is a labor-intensive task. Empirical evidence that BERT-based models perform well for other text classification tasks has motivated our automated approach for causal sentence prediction. This study presented LERCause, a baseline tool for causal sentence identification in the nuclear safety domain, including an annotated LER dataset and BERT-based models. To our knowledge, this is the first attempt to deploy pre-trained language models for extracting causal sentences from nuclear event reports.

Our experiments used three BERT-oriented approaches (BERT, BioBERT, and SciBERT). We compared their accuracy against a heuristic method, three traditional machine learning techniques (LR, GB, and SVM), and a CNN. All these models were incorporated into our benchmark tool, LERCause. Among the tested classifiers, BioBERT performed best, with an F1 score of 94.49%. In the experiment on the impact of dataset size on model prediction accuracy, BERT-based models showed stable performances with small-sized samples. Our shared code and dataset can serve as a baseline to validate other causal sentences extraction techniques and develop novel approaches in future research.
